# Cultural beliefs and practices on perinatal death: a qualitative study among the Lango community in Northern Uganda

**DOI:** 10.1186/s12884-023-05550-4

**Published:** 2023-04-03

**Authors:** Anna Agnes Ojok Arach, Noeline Nakasujja, Joseph Rujumba, David Mukunya, Beatrice Odongkara, Milton W. Musaba, Agnes Napyo, James K. Tumwine, Victoria Nankabirwa, Grace Ndeezi, Juliet Kiguli

**Affiliations:** 1 Department of Nursing and Midwifery, Faculty of Health Sciences, Lira University, P.O Box 1035, Lira, Uganda; 2 Department of Paediatrics and Child Health, School of Medicine, Makerere University College of Health Sciences, Kampala, Uganda; 3 Department of Psychiatry, School of Medicine, Makerere University College of Health Sciences, Kampala, Uganda; 4 Department of Public Health, Busitema University Faculty of Health Sciences, Mbale, Uganda; 5 Department of Paediatrics and Child Health, Gulu University Faculty of Medicine, Gulu, Uganda; 6 Department of Obstetrics and Gynaecology, Busitema University Faculty of Health Sciences, Mbale, Uganda; 7 Department of Epidemiology and Biostatistics, School of Public Health, Makerere University College of Health Sciences, Kampala, Uganda; 8 Centre for Intervention Science and Maternal Child Health (CISMAC), Centre for International Health, University of Bergen, Bergen, Norway; 9 Department of Community Health and Behavioural Sciences, School of Public Health, Makerere University College of Health Sciences, Kampala, Uganda

**Keywords:** Culture, Perspectives, Beliefs, Practices, Stillbirth, Perinatal death, Uganda

## Abstract

**Background:**

Perinatal death has profound psychosocial effects on women and their families. Sociocultural contexts influence the burden, rituals and bereaved’s support. Little is known about cultural beliefs and practices related to perinatal death. This study explored the cultural perspectives of the Lango community on perinatal death.

**Methods:**

This study utilised a focused ethnographic design anchored on a symbolic interactionist framework to understand the meanings attached to beliefs and practices on stillbirth or neonatal death among the Lango community in Lira District, Northern Uganda. Participants were sampled purposively for FGD while key informants were identified through snowballing technique. Data were audio recorded in Lango, transcribed, and later translated, a codebook was developed and data entered into Atlas. ti version 8.4.26 and then coded. It was analysed both deductively and inductively into themes.

**Results:**

Stillbirth and early neonatal death both attract similar rituals as would an older child. Burial is not rushed and is attended by family members and close friends. Stillbirths and children that die before naming are buried without names. Bereaved families are comforted and encouraged about future pregnancies. Currently, Lango associates the deaths to biomedical explanations such as teenage pregnancies, inadequate pregnancy care, health system challenges and poor health-seeking behaviour, unlike previously when they were attributed to consequences of unacceptable social behaviours, superstitious beliefs and witchcraft. Antenatal care and health facility childbirths are currently preferred over traditional practices for good pregnancy outcomes.

**Conclusion:**

Stillbirth or early neonatal death is viewed as the death of a child, different from other settings. Thus, rituals are performed to honour, create memory, and maintain the connection with deceased babies. Bereaved parents are supported. Health care workers need to provide culturally sensitive support to parents after perinatal loss. The prevailing beliefs of perinatal death cause in terms of biomedical explanations consistent with known determinants and preference for health facility care for prevention creates an opportunity for improving perinatal health.

## Background

The risk of perinatal death in low and middle-income countries remains a challenge, with an estimated 43 deaths per 1,000 births in Africa [1]. The contribution of cultural context is a recognisable factor in reducing these deaths and preventing the negative consequences. Although, events and behaviours following perinatal deaths depend on cultural context [2–7], “limited attempts have been made to explore the cultural context of perinatal loss” [8].

Moreover, perinatal death terminologies vary across cultural settings, and influence community perceptions about causes, mourning and burial rituals. For instance, in some communities in Ethiopia, Ghana, Nepal and Taiwan, perinatal deaths are viewed as non-human or natural occurrences with immediate burials in restricted private places or managed by hospitals with parents unaware of the procedure [9–12]. Perinatal deaths are attributed to supernatural powers, and past deeds and mourning are prohibited with an exception of private rituals held in Taiwan. This is different from other contexts such as Western countries where stillborn babies are valued, openly discussed and funerals held [11, 13–17].

Rituals related to maintaining the deceased’s memories are reported as a form of psychosocial support and aid in coping [9, 11]. It varies with culture and depends on the meaning of death [18]. Bereavement experts note that acknowledging a loss through the participation of parents in rituals facilitates emotional expression and, thus, healing and recovery [19]. Although the use of rituals and symbols such as spending time with stillborn babies and creating memories during bereavement have been widely studied in western countries [11, 13–17], little is published from the African continent [2, 12, 20, 21]. Moreover, sub-Saharan Africa has the highest burden of perinatal deaths, but with varied cultural beliefs and practices.

In Uganda, about 4% pregnancies end in perinatal deaths [22], however limited studies highlight the cultural context yet there are varied ethnic groups and cultural practices. For example, in some regions stillborn babies are not perceived as persons thus, immediate burial are secretly held with some parents not seeing or holding their deceased [2]. Bereaved women mourn in silence with no health system support [20]. Stillbirths are attributed to spiritual, biomedical, social and supernatural beliefs [2, 20]. Northern Uganda with high perinatal mortality of 43 deaths per 1000 births [23] is less researched yet sociocultural context influences burden and care after perinatal death [6, 7, 24], and perinatal death varies within and between countries [25, 26]. Thus, awareness of the local beliefs and practices from this region will enable provision of culturally sensitive support and targeted prevention interventions. Further, since majority of past studies are from the bereaved parents’ perspectives with less representation of the cultural leaders who are decision-makers on health care matters, there is need to understand their perceptions. This study explored the cultural perspectives of the Lango community in Northern Uganda on perinatal death.

The theory of symbolic interactionism has been used to explain the meanings and actions regarding stillbirth or perinatal death. Symbolic interactionism, coined by Herbert Blumer (1900–1987) from the teachings of George Herbert Mead, focuses on explaining human behaviour in terms of meanings learned through social interactions. Herbert Blumer based on three premises that is, human beings act based on the meanings they assign to things; the meanings are given based on social interactions, and lastly, the meanings given are not permanent [27]. Therefore, the theory can be used in naturalist inquiry to get meanings attached to symbols in this case rituals, practices and behaviours following a perinatal death from the native population.

## Methods

### Study design and setting

We conducted a focused ethnographic study [28] aimed at describing and interpreting the cultural beliefs, practices and behaviours of the Lango community in Northern Uganda on perinatal death. The design enabled a health care research with focus on Lango culture about perinatal death and the findings are expected to have relevant application to reducing perinatal death.

The study was conducted in Aromo, Ogur and Agweng sub-counties of Lira, one of the nine Lango sub-region districts found in Northern Uganda. The Langi, the dominant ethnic group in the sub-region, speaks *Lango* and are mainly subsistence farmers. The Langi are said to have migrated alongside other Nilotic groups from Ethiopia and settled in the Lango sub-region about 400 years ago [29]. Currently, they occupy the present districts of Lira, Apac, Kwania, Oyam, Kole, Otuke, Alebtong, Dokolo and Amolatar in central-Northern Uganda. They are neighboured by the Acholi, Iteso, Karamojong, Banyoro and Baganda in the northern, eastern, western and southern boundaries. Some of these ethnic groups are also living in the sub-region. Currently, there are 153 clans each with a hierarchical leadership ranging from the lowest (*won pacu*) to the highest, the Clan head (*Awiitong*). All the clan heads are united under the Lango cultural foundation with a paramount chief (Won *nyaci*) elected in 2005 [29]. Clan relations are strong, and intra-marriage is prohibited. Each clan has social norms, values and rituals which must be followed. According to Epila-Otara, each clan has taboos which are commonly used to maintain morals, discipline and good health, especially during childbearing age [29]. Knowledge and skills on various aspects of life are transferred verbally to young children using folktales, riddles, plays and proverbs.

Families live in scattered settlements or groups. It is common to have extended families within a homestead (*Doggola*) for security, and social protection of the homes and this conforms to blood relations. Also, farming and other activities, such as celebrations and mourning, are communal. Childbearing especially a male child is highly valued among the Langi, because through him the family lineage is preserved [30]. As written by Epila-Otara, the Langi are reserved and cautious when it comes to procreation [29]. Childbirth is very important, and no preparation, such as making a baby strap, is made because of the fear of either losing a baby at birth or delivering multiple babies. Preparation or uttering comments is associated with a curse. Lira district was identified for this study based on locally observed poor maternal and neonatal indicators. The presence of the Survival Pluss trial previously implemented in the area, also motivated the district's choice [31].

### Participants and sampling

The participants were female and male clan and opinion leaders. Their roles as guardians and promoters of cultural beliefs and practices in the Lango community meant they would supply rich information on the cultural beliefs and practices.

They were recruited in the study if they belonged to Lango ethnic group, were older, between the age range of 50 to 80 and lived in Lango-sub region mainly rural areas. This age range was meant to give experienced and knowledgeable persons who have lived through the past and the current cultural periods. They were excluded if sick at the time of the study and unable to consent.

Participants for focus group discussions were purposively identified through the community leaders and village health teams while the key informants were located using the snowball technique, that is, participants interviewed directed research team to other potential participants. Whenever an individual was suggested, the study team comprised of the first author and an assistant traced his or her home using telephone contacts or directions by people. Participants were then invited to participate in the study if they fulfilled the criteria. During the invitation, they were informed about their voluntary participation and that they could choose to withdraw from the study at any time if they wished.

### Definition of operational terms

Perinatal death comprises of stillbirth and early neonatal death.

Stillbirth is defined as the birth of a stillborn baby from 28 weeks of pregnancy.

Early neonatal death is the death of a newborn baby within 7 days of life.

### Data collection

The data collection were conducted by a nurse researcher (1^st^ author) and two research assistants all natives of Lango sub-region using focus group discussions (FGDs) and key informant interviews (KIIs). Before each interview, participants and the data collection team were self-introduced. Participants who were mainly clan leaders below the clan head *(Rwot*, *Jago*, women representatives and advisors) took part in FGDs. Six focus group discussions, three for males and three for females, comprising of 6 to 8 members each, were conducted between November and December 2020. Six FGDs with 6 to 8 participants were found sufficient as recommended [32, 33]. The focus group meetings were held in the council hall of Aromo sub-county and a primary school classroom in Agweng Town Board. Further more, 13 key informant interviews were conducted with clan heads (*Awii tong*) and advisors affiliated to the Lango Cultural Foundation between December 2020 and January 2021. The KIIs were held at the participants’ homes. Permission was sought for audio recording. We conducted all the FGDs, and KIIs in *Lango* with a semi-structured interview guide previously developed, translated and pre-tested on a few members of the community. Participants were asked questions on the meaning of perinatal death, perceived causes and preventive measures, care and support, and mourning and burial practices for stillbirth and early neonatal death. Where necessary, probing was done to understand more about the topic areas. Since cultural meanings attached to symbols change with social interactions and time, considerations were made to the present and past beliefs and practices. Interviews and FGD sessions were audio recorded with a recording device and non-verbal interactions observed and noted. On average FGDs lasted between 1 to 2 h, and KIIs between half to an hour as recommended [32–34]. Sociodemographic information was collected during appointment scheduling, on the day of interviews and during reference check after data collection. After each interview or group meeting, a debrief was held to discuss the everyday expressions, unique features observed, and issues that needed follow-up in subsequent interviews. In both FGDs and KIIs, interviews were conducted until we reached saturation, that is, there was no new information arising on the different sub-topic areas from addition of participants/groups [35].

### Research team and reflexivity

The data collection team, especially the first author and her assistants, are Langi, who have lived in the Lango sub-region for most of their lives. They have participated in and witnessed some beliefs and practices related to pregnancy and childbirth, stillbirth or children born alive and dying within a week after birth from their close families and previous studies in the study area. With this background, the data collection team set aside their knowledge to learn the meanings of the things Langi say and do about losing children around birth and one week old. The participants felt they were teaching the young Langi who wanted to learn about their culture (*Tekwaro*). It is possible that some of the “sayings” and their meanings could have been taken for granted, having been “an insider” of the Lango community and a medical professional. The senior co-authors, specialised as medical anthropologists who were “outsiders” to the culture, helped identify the gaps that were filled during the data analysis and report writing.

### Data analysis

Data were analysed using the thematic content approach. Before analysis, we listened to audio records more than once and transcribed them verbatim in Lango. The Lango transcripts were then translated into English by an independent native fluent in English. Each transcript was cross-checked with the audio record to ensure accuracy, consistency and completeness of the data collected. Clarification and checking of the responses were made with some focus group participants during prelimary analysis. We focused on the perceived cause, prevention, mourning and burial rituals related to perinatal death. Codes were assigned to important text information using open and list codes. Consideration was made to both the manifest and latent content [36]. Similar codes were grouped into categories, and categories were put together to form themes [37]. Some of the codes and themes were formed a priori [38]. Data coding and analysis were conducted with the aid of ATLAS.ti 8.4.26 [39]. The whole process of analysis was carried out by the researcher (1^st^ author) and research assistants, then later verified by senior co-authors. Participants’ quotes were used to support the results.

## Results

Forty-eight people with ages ranging from 50 to 90 years participated in the study. The majority were females, 52% (25), married 58% (28), and attained at least primary education 38% or secondary education 33%. Those with no formal education had undergone comprehensive Christian instructions during their teenage years. They had also experienced a perinatal death from a biological or a grandchild. The participant demographic characteristics are found in Table 1.

**Table 1:** Sociodemographic characteristics of the study participants

Characteristics	n (%)
Sex	
Male	23 (48)
Female	25 (52)
Age	
50 - 60 years	23 (48)
61 - 70 years	13 (27)
Above 70 years	12 (25)
Marital status	
Married	28 (58)
Widowed	20 (42)
Level of Education	
No education	14 (29)
Primary	18 (38)
Secondary and above	16 (33)
**Total**	**48 (100)**

**Fig. 1:**
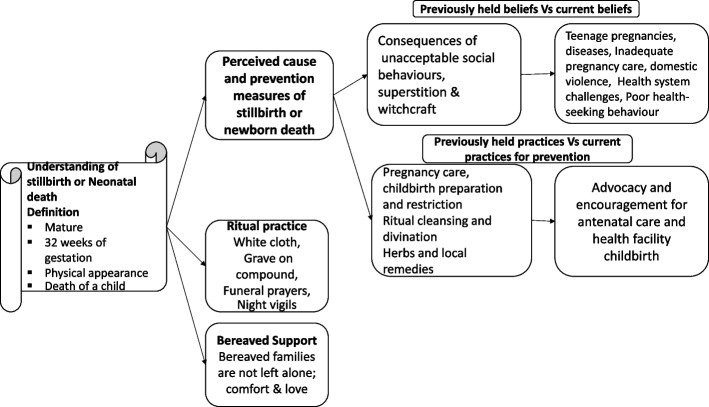
Perspectives, beliefs and practices on perinatal death

The detailed explanation of the themes that emerged during the analysis are displayed below and also presented in Fig. 1.

### Theme 1: understanding of stillbirth or early neonatal death

This community views stillbirth or early neonatal death as death of a child, similar to an older child. Particpants defined stillbirth as a mature baby born with no breathing or movements after eight months of pregnancy and early neonatal death as one born alive and dies soon after birth. They look for signs of a fully developed and mature baby in order to differentiate from a miscarriage. Maturity is expressed in terms of pregnancy duration ranging from 8 months onwards, body parts fully developed. For instance, a male is said to be mature when his genitals are dark in colour. In addition, they look for physical appearances such as skin macerations, rotten umbilical cords or congenital malformations. The deceased baby is viewed as a member of the family.

### Theme 2: previous held beliefs versus current beliefs about child death

The past beliefs on causes of child death were centered around consequences of past deeds, witchraft and superstition, while the current beliefs involved biomedical causes as stipulated below.

### Sub-theme 1: past beliefs about the cause of perinatal death

#### Consequence of unacceptable social behaviour

Participants reported that the death of a newborn child was in the past believed to result from socially unacceptable behaviours of the bereaved parents. The reported behaviours are adultery, unnecessary oath (*kir*), incest and violation of taboos. For instance, to have a successful childbirth, a woman who committed adultery made a public confession in the presence of the birth attendant and close elderly female family members who are allowed in the labour room. Other bad behaviours, such as theft and lack of respect for elders by the pregnant women, attracted curses and lamentations (*lamere*) such as “you will never hold a baby” resulting in perinatal deaths.

“If an elder wanted to educate a daughter–in–law and she began to insult him. ……., could the elder be happy with her? …. He wouldn’t curse openly, but in his heart, he had already made a curse as he was terribly angered and sometimes did not eat food brought to him, Wasn’t that not a curse already?” 77 year old male FGD_02.

#### Witchcraft and superstition

Witchcraft and evil eyes resulting from hatred and jealousy of other people were believed to contribute to death. Key informants reported that women believed some people “saw them with evil eyes”, implying the jealous people had bad intentions and wished for adverse pregnancy outcomes. The “witchcraft (*tim jojok*)” is believed to be an evil activity done using supernatural powers by unidentified persons resulting in perinatal death. Sometimes there were attacks by malevolent spirits (*cen a dano otoo or aping*) that killed the baby in utero. They reported that the attacks would occur when the spirits were not pleased with the actions of the living family members.

“Sometimes, anything that belongs to a baby could be removed for evil purposes. When this happened, it would cause the death of the child. Death occurring this way is blamed on an older woman suspected of practising witchcraft. Therefore, the death of only one child is considered bad luck, but when two or three children die in a series, the danger is suspected”. 69 year old female Key Informant (KI)_12.

### Sub-theme 2: current beliefs held about the cause of child death

#### Domestic violence and insufficient care of pregnant women

Participants felt there is increased sexual and gender-based violence on pregnant women perpetrated by their partners, with several cases witnessed by them. In addition, the partners are not taking good care of the pregnant women. Study participants felt that pregnant women lack “proper nutrition” and are not receiving enough support for antenatal care.

“There are some men who drink too much and when they go back home, they start a fight with their wives who had been left at home, that thing brings about the death of many babies”. 54 year old male FGD_04.

#### Teenage pregnancies and diseases

Participants reported common cases of teenage pregnancies and diseases compared to the past. The commonest diseases are malaria and sexually transmitted infections. Malaria was mentioned as a problem concerning the late seeking of care, while STIs were blamed on multiple sexual partners of the husband. In all the interviews, teenage pregnancies and early marriages were stressed as the major contributors to losing babies around birth. This is contrary to the past when girls and boys got married when considered as mature, 20 years and above. Other causes include drug abuse during pregnancy, use of modern contraceptives, cord complications, minimal birth spacing and abnormal positioning of the foetus.

“There are young girls who start getting involved in sexual relationships very early, then they join family planning to avoid pregnancy and it reaches a time they conceive when still young. They do not follow instructions given to them by health workers. And by the time she is giving birth, she can’t deliver from a Health Center III”. 56 year old male FGD_06.

#### Health system challenges and poor health-seeking behaviour

The current health system challenges reported include health care providers’ behaviours such as delayed attending to women, harassment of the women during labour, and great distance between households and health centres. Moreover, these days people are encouraged to seek health facility care. Participants noted that other women poorly attend antenatal care and others prefer home childbirth. Participants mainly from the focused groups acknowledged the unskilled or inexperienced birth attendants as contributing to the child deaths.

“….., most problems are irresponsibility of the midwives or medical personnel. Imagine how they talk to a mother in labour, “You are pretending,………, you think who will come and help you if you do not push the baby” They leave the woman there alone, …”. 58 year old male KI_06.

“Women don’t want to go to the Health Centre because of their problems which are the means of transport, which is kind of challenging these days”. 50 year old female FGD_05.

#### Poverty and religious beliefs

Participants mentioned poverty as contributing to the deaths. For example, a key informant expressed that whereas in the current time, most of the health care is left to the health system to manage, people cannot afford the cost of transport to health facilities and treatment. Participants also reported that child death is the plan of God.

“Poverty is causing us death because if a woman is in labour pain and should be referred to another health facility, hiring a vehicle is about 100,000 UGX and above! At the referral centre, requirements are needed yet there is no money. People budget and decline to go”. 55 year old female FGD_03.

### Theme 3: previously conducted prevention practices versus current practices

The previous held practices for alleviation of sickness and prevention of child death involved care of pregnant women, childbirth preparation and restriction, use of traditional herbs and local remedies, ritual cleansing and divine support while the current measures are centred on antenatal care and health facility childbirth as follows;

### Sub-theme 1: previous practice to prevent child deaths

#### Care for the pregnant woman

Participants reported that pregnant women were accorded comprehensive care. In this, they meant the well-being of pregnant women was well taken care of by ensuring good nutrition and a hygienic and stress-free environment. The foods were mainly green vegetables, cereals and fruits. The women were exempted from hard work in late pregnancy. Close attention was provided to the pregnant women by their mothers-in-law unlike these days when they feel women are left in the care of their husbands and health care providers.

“…., women were taken care of by ensuring that they were healthy when pregnant. When she had conceived; she was given food varieties; ensuring that she did not do heavy work, not stressed up and also stopping her from journeying unnecessarily to protect mother and unborn baby from people with bad intentions”. 75 year old female FGD_01.

#### Childbirth preparation and restriction

The importance of birth preparation and restriction which was done was emphasized. Mother-in-law prepared the required materials for the birth and also informed the birth attendant in the village. This was done without the pregnant woman's knowledge because of the Lango proverb “*Pe ikwoo abeno abongo atin*” (you do not make the carrier for the baby before its birth). Participants felt the woman in labour was well taken care of, although it was a home birth. The birthing process and the newborn baby were concealed from visitors and individuals suspected to practice witchraft using a symbol of a fresh tree branch (*oduggu*) erected at the compound entrance. Cultural beliefs and norms were adhered to during and after birth.

“…., the child would be protected very securely. If it is a boy child it would be the mother-in-law to bathe him for three days, and a girl for four days”. 75 year old female KI_13.

#### Traditional herbs and local remedies

Health problems of pregnant women were managed using herbs or locally available remedies at home. The mothers-in-law were knowledgeable of the herbs sourced from the nearby bushes. Sometimes treatment was sought from a traditional herbalist. Family elders performed incantations (*Lemu*) for suspected persons behind the deaths. This was meant to scare away the bad individuals responsible for the deaths in a family.

“There was a medicine that we use to give pregnant mothers with waist pains. It was dug from “dago” and the medicine was called “odoro”. When this herb was given to the woman feeling waist pain, the pain would stop immediately”. 75 year old female KI_13.

#### Ritual cleansing and divine support

There were always several attempts when seeking healing or prevention of child deaths. If the herbs were non-effective, rituals and divine solutions were sought for the woman. If a woman experienced 2 to 3 perinatal losses ritual cleansing was performed for the preceding baby born alive, on the day of bringing him/her outside the house. The ritual involved naming the baby with names outside the clan, passing him/her between two women under a barn and pricking the ears. This was done by 2 elderly women from different clans. The baby became a ritual baby *(atin akwera*), evidenced by the name and ears pricked. These were interpreted as prayers meant to reverse or avoid curses suspected to have caused the previous deaths. If the baby died again, the divine solution was sought from a spirit medium.

### Sub-theme 2: current prevention practices for perinatal deaths

#### Health facility care currently recommended

Participants reported that currently, people are encouraged to seek antenatal care and have childbirth in health facilities where skilled birth attendants manage them. These cultural leaders feel the Lango traditional practices have been taken over by modern medicine and western religion. Moreover, this religion also encourages health facility care. They suggested the partners need to support pregnant women in seeking health care, and the health care providers are encouraged to serve with a kind heart. Mothers-in-law are encouraged to support their daughters in accessing health care during pregnancy and childbirth.

“…..no one must get pregnant and keep at home. She must go for an antenatal check-up. Then the women must not go alone, so it is our responsibility to ensure we force these men to go together with their wives for antenatal check-ups”. 57 year old male FGD_04.

### Theme 4: rituals for honour and memory of a deceased child

The rituals held for burial of a stillborn baby and a newborn death are similar to that of an older child or an adult. The practice have had minimal changes as described below;

#### Burial with prayers to bid farewell to the child

The death of a child is made known, and extended family members and close friends attend the burial. Burial is conducted after a night or two depending on the availability of burial resources and key persons such as church ministers and maternal relatives. Stillborn babies and those who die before naming are buried without names. Prayers are held during the burial as was conducted in the past. However, currently church ministers lead the funeral prayers while in the past it was led by a group of elders called *Itogo *(experienced, respected, wise leaders who made decisions and interceded with God (*jojok amalu*)) on behalf of the group. These are all because of the belief that one continues to live after death. Therefore, collective prayers are meant to ask God to receive the deceased and bid farewell to the soul. Rest well (*but i kuc*) go in peace (*wot i kuc*); were commonly recited in the past, while in present times, the prayers are structured services led by church ministers. The prayers are also intended for the comfort of the bereaved persons.

#### White cloth as a sign of love and holiness

Like adults, the deceased babies are cleaned, dressed in the most beautiful clothing, and wrapped in white cloth before burial. While cleaning and dressing, elderly women observe the deceased baby to rule out deformities and other detectable causes of death. Cleaning and dressing mean that a person going on a journey, transiting to another world or returning home to meet God (*Jok amalu*) must be clean and smart. According to participants, these procedures show love and is appeasing to the deceased’s soul. White clothing is associated with holiness moreover, the deceased baby is considered innocent and sinless, therefore, the journey back to God is without any impediment. Red or black cloth is not allowed because red is dangerous and evil. An informant emphasised as stated below;

“………………..white represents cleanliness or holiness. So, the white colour gives the feeling that the person is going to a holy place. Anything red is also avoided because it is associated with blood”. 72 year old female KI_01.

#### Grave on the compound as part of the family

Burial site is located at the home of a paternal relative (grand father). The grave is dug at the coumpound side with the deceased’s head facing home. In the past, the grave was marked using four stems of *oduggu* but these days, families attempt to cement it. These practices have remained over time and were mentioned by all the participants. Burial in the compound means that the deceased remains part of the family, and, therefore, peace is maintained. Doing the contrary would imply the person is neglected, deserted, and not loved, thus angers the deceased, resulting in problems or subsequent deaths. A participant was quoted saying….

“They face the head of the baby home so that when its soul is coming, it says that this is home. If the head faces the bush, it brings misfortunes and deaths in the family that the living people hate him /her”. 77 year old male FGD_06.

#### Night vigils for mourning and guarding the deceased

Participants reported that there is an old practice of keeping a night vigil for 3 or 4 days which commences immediately after burial. For deceased males, it takes three days, while for females, it last four days. This number of days corresponds to the period a newborn is kept indoors after birth. The vigil comprises a bonfire (*gadi*) set in the middle of the compound and burns continuously (both day and night). It is not for cooking but is meant to light the area and symbolises mourning. With exception of children and pregnant women, adults sleep around it at night. Sleeping in the open is meant for guarding the deceased body. During this time, people, especially older women, give comforting and encouraging messages to the bereaved parents. After the recommended days are complete, an elder female relative collects ash early in the morning and disposes off with care (*coko buru*), followed by pouring cold water on the fire site to calm the family and restore peace. The ash collection was reported as the official end of mourning.

“There is always a bonfire where people gather and drink, and eat together. Even when the child is only one week old, it is considered a person, therefore necessitating this ceremony. After the recommended days, the ash from the fire they used is removed together and then they disperse. The ash is taken to a swamp and disposed of with great care”. 72 year old female KI_01.

### Theme 5: measures for the bereaved support

Death of a child, according to participants, means a loss to the family and the clan. The following were commonly mentioned as actions to support the bereaved in adapting to the loss:

#### Families who have lost a child are not left alone

Participants reported that traditionally when one has lost a child, relatives and friends stay physically close to the bereaved, showing sympathy and comforting them with good messages such as death happens to everyone. Currently, verses from the holy books and gospel songs are used for consolation. In addition, church ministers and members, remain close to the bereaved. Similar past experiences are shared, insensitive comments are restricted, and the lady is relieved of house chores. This is meant to encourage and comfort the bereaved so they do not feel left alone. An FGD participant stressed that discussion of issues non-related to death (distraction) is common during bereavement because it promotes mental relaxation.

“True Langi with their attitude of love towards one another whenever death occurs, usually leave whatever they are doing and camp at the home of the bereaved, staying close and eating from there. The bereaved mother is counselled by elderly women…, they sit close to her and she is not allowed to do any chores”. 83 year old male KI_04.

Both FGD participants and key informants reported that when a person loses a loved one, clan members, friends and relatives support the burial arrangement. They make a financial and material collection to support the bereaved in carrying out the burial. The support is to show love for the bereaved.

“Whether it was the death of a baby or an adult, the bereaved did not cook. It was the neighbours who would cook and take food to the family where death had occurred. That was the first thing to calm down the family members”. 83 year old male KI_04.

#### The bereaved couple are encouraged to plan for the next pregnancy

Parents who have lost a child are encouraged to produce another child. They use the proverb, “if the mother stone has remained, the daughter stone can still be got (*ka min kidi odong, nyare nwongere*). The mother and daughter stones concept is used to imply the woman and her newborn baby. The concept originated from home grinding stones used for grinding millet and other cereals. Participants stated that the proverb is used to comfort and encourage the couple to forget about the loss and have another pregnancy. They also say, “*nyuka ka oton i cip pe nangere” (*when porridge spill on dirty linen, it cannot be licked). Both proverbs are meant to encourage anticipation of the next pregnancy rather than worry about the loss of their baby.

“You take heart and stay, even when the child has died, there is no cause of alarm as long as the mother stone is left because when the mother stone is alive, you can get another daughter stone and still be able to continue grinding”. 58 year old female KI_10.

## Discussion

This study explored the cultural perspective of the Lango community in Northern Uganda on perinatal death. The findings showed that stillbirth or death of a liveborn baby is viewed as the death of a child. In the past, these deaths were believed as a result of unacceptable social behaviours, superstitions and witchcraft but currently, they associate the deaths with biomedical causes. In terms of practices, this community sought traditional and local measures while currently, they prefer health facility care. The deceased babies are honoured and memorised through rituals. Bereaved families are supported to cope with the loss.

The meaning attached to stillbirth and early neonatal death as a child implies the values held which are similar to Afghanistan, though contrary to those from other regions in Uganda, Ethiopia, Taiwan and Ghana where stillbirths are viewed as non-humans evidenced by the invisible mourning and rituals following the deaths [2, 9, 10, 12, 20]. Participants’ value for the deceased could be due to their belief in life after death. Their past belief and practices could potentially be explained by the prevailing traditional beliefs and scarcity of health care services at that time. Although their intentions were to prevent adverse perinatal outcomes, the perceptions are likely to deter families from seeking appropriate biomedical care. Similar findings have been reported in Pakistan, Cameroon, Liberia, Ghana and Uganda [20, 40–44] where cultural beliefs are still present and affecting pregnancy and utilization of health facility for childbirth.

Our findings revealed a shift in cultural beliefs with most people associating such deaths with biomedical explanations. The shift could be explained by social interactions of the community with systems like modern medicine, western religion and education that changed their beliefs. Moreover, the traumatizing effects, absence of a medical diagnosis and having to declare “what led to death” during burials in the study area motivate search for the cause as was also noted in India [45].

Association of perinatal deaths to biomedical explanations as was common in this study, has been noted in India, Nigeria, Ethiopia, Uganda [12, 20, 45, 46], however varying from other rural communities in Nigeria, Ethiopia where socio-cultural practices are still predominantly common during the perinatal period [47, 48]. Factors such as malaria, sexually transmitted infections, health system challenges, poor health seeking behaviour, teenage pregnancies and domestic violence mentioned by participants reflect their knowledge on the determinants of perinatal death. Thus, awareness of a native’s knowledge and beliefs is able to guide health managers in designing programs for reducing perinatal mortality.

Currently, this community emphasises health facility care for issues related to pregnancy and childbirth, a practice consistent with biomedical care. This could be because of the continuous encouragement to utilise health facility care coupled with abolished action of traditional birth attendants in the country. Therefore, the positive support of health facility services gives opportunity for improving perinatal health.

The rituals for honouring and keeping the memory of the deceased babies enables the bereaved parents and families to accept and come to terms with the loss, and stay connected with the baby; a way of recognizing and validating the loss. Varying rituals and symbols with similar meanings have been noted in both developed and developing countries alike, with positive impact on coping [11, 14, 16, 49, 50]. Full participation of friends and relatives in these rituals is said to justify the love and support for the bereaved, thereby comforting them. Whereas rituals are held publicly in this community similar to western countries, other contexts are private with no memories [2, 9, 20] and in some communities in Afghanistan, Ghana and Ethiopia, funeral rites are uncommon. Therefore, as was noted by the previous authors [6, 51, 52], health care workers need to provide culturally sensitive support to parents after perinatal loss.

Being close and supporting the bereaved parents in this study symbolises love, recognition and sharing in the loss; potentially explained by the communal way of life where events such as deaths and celebrations are managed as a group, symbolising unity. Family support has been attributed to healing [49]. Open discussion about perinatal death noted in this study is restricted in Uganda, Ethiopia, and Ghana [2, 20]. The practice of support for the bereaved parents observed in this community have also been noted in both developed and developing countries [2, 8, 11, 21, 53, 54]. Thus, the need for always giving support to parents after perinatal loss.

### Strengths and limitations

Past studies have explored experiences of perinatal death from the parents’ perspective; however, few have considered the elders who promote and encourage adherence to cultural beliefs and practices. This study provides views of cultural leaders and elders on the meanings attached to perinatal death from the Lango community perspective. The inclusion of male and female leaders in this study corresponds to the different gender roles concerning cultural beliefs and practices. The data collection team are native to the study area; therefore, they were able to give a good description of the culture under study. Culture is dynamic; therefore, attempts were made to explore current and past beliefs and practices. The application of this study findings to other settings with different socio-cultural characteristics may be limited. Although participant observation as a data collection method was not fully utilised as the focus was on exploring the present and past cultural perspectives of the persons who have lived it, experiences of the study team from the burial rituals and similar events they attended from the study area and their close families contributed to their knowledge on perinatal death.

## Conclusion

This community views stillbirth or early neonatal death as deaths, different from other settings. Currently, they perceive perinatal deaths in terms of biomedical explanations, consistent with known determinants of perinatal death and advocates for health facility care over traditional practices for prevention. Thus, an opportunity for designing health programs for improving perinatal health. Rituals are used in this community to honour, connect and keep the memory of the deceased babies while comforting the bereaved. Therefore, health care providers need to provide culturally sensitive support to parents after perinatal loss.

## Data Availability

The datasets generated and analysed during the study are not publicly available as the data cannot be anonymised adequately for public sharing. The data may be available from the corresponding author on a reasonable request.

## Abbreviations

FGD: Focus Group Discussion
KII: Key informant interviews
